# Household transmission of SARS‐CoV‐2 during the Omicron wave in Shanghai, China: A case‐ascertained study

**DOI:** 10.1111/irv.13097

**Published:** 2023-02-05

**Authors:** Zhongqiu Wei, Wenjie Ma, Zhonglin Wang, Jingjing Li, Xiaomin Fu, Hailing Chang, Yue Qiu, He Tian, Yanfeng Zhu, Aimei Xia, Qianhui Wu, Gongbao Liu, Xiaowen Zhai, Xiaobo Zhang, Yan Wang, Mei Zeng

**Affiliations:** ^1^ Department of Infectious Disease Children's Hospital of Fudan University Shanghai China; ^2^ School of Public Health Fudan University, Key Laboratory of Public Health Safety, Ministry of Education Shanghai China; ^3^ Department of Medicine Children's Hospital of Fudan University Shanghai China; ^4^ Department of Hematology and Oncology Children's Hospital of Fudan University Shanghai China; ^5^ Department of Respiratory Medicine Children's Hospital of Fudan University Shanghai China; ^6^ Shanghai Institute of Infectious Disease and Biosecurity Fudan University Shanghai China

**Keywords:** household transmission, Omicron variant, SARS‐CoV‐2, vaccine effectiveness

## Abstract

**Objectives:**

We used a case‐ascertained study to determine the features of household transmission of SARS‐CoV‐2 Omicron variant in Shanghai, China.

**Methods:**

In April 2022, we carried out a household transmission study from 309 households of 335 SARS‐CoV‐2 pediatric cases referred to a designated tertiary Children's Hospital. The detailed information can be collected from the 297 households for estimating the transmission parameters. The 236 households were qualified for estimating the secondary infection attack rates (SAR_I_) and secondary clinical attack rates (SAR_C_) among adult household contacts, characterizing the transmission heterogeneities in infectivity and susceptibility, and assessing the vaccine effectiveness.

**Results:**

We estimated the mean incubation period and serial interval of Omicron variant to be 4.6 ± 2.1 and 3.9 ± 3.7 days, respectively, with 57.2% of the transmission events occurring at the presymptomatic phase. The overall SAR_I_ and SAR_C_ among adult household contacts were 77.11% (95% confidence interval [CI]: 73.58%–80.63%) and 67.03% (63.09%–70.98%). We found higher household susceptibility in females. Infectivity was not significantly different between children and adults and symptomatic and asymptomatic cases. Two‐dose and booster‐dose of inactivated COVID‐19 vaccination were 14.8% (5.8%–22.9%) and 18.9% (9.0%–27.7%) effective against Omicron infection and 21.5% (10.4%–31.2%) and 24.3% (12.3%–34.7%) effective against the symptomatic disease.

**Conclusions:**

We found high household transmission during the Omicron wave in Shanghai due to presymptomatic and asymptomatic transmission despite implementation of strict interventions, indicating the importance of early detection and timely isolation of SARS‐CoV‐2 infections. Marginal effectiveness of inactivated vaccines against Omicron infection poses a great challenge for outbreak containment.

## 1 INTRODUCTION

The COVID‐19 pandemic caused by SARS‐CoV‐2 has resulted in unprecedented global health crisis and more than six million deaths worldwide since December 2019.^1^ Despite the increasing natural immunity and vaccine‐induced immunity are common in population, the newly emerged Omicron variant, with increased transmissibility and immune escape properties, has rapidly replaced previous strains and driven a new surge of SARS‐CoV‐2 infections across the world.^2^, ^3^ China maintained local containment through effective border controls and non‐pharmaceutical interventions (NPIs) since 2020 and has successfully coped with several importation‐linked local outbreaks of SARS‐CoV‐2 variants.^4^ In the meantime, Chinese government spared no efforts to promote countrywide mass COVID‐19 vaccination roll‐out among adults since April 2021 and among children aged 3–17 years since July 2021.^5^, ^6^ Nevertheless, following the first cluster of Omicron infections detected in late February, 2022, a local epidemic wave caused by Omicron BA.2 sub‐lineage hit Shanghai, one of the largest metropolitans with a population of over 24 million in China. Due to the previous success dynamic zero containment policy implemented at national level, Shanghai had never experienced natural outbreak of COVID‐19 since April 2020. Thus, the population immunity induced by prior SARS‐CoV‐2 infection was lacking. As of March 22, 2022, more than 90% and 45% of the individuals in Shanghai have completed primary and booster doses of COVID‐19 vaccination, respectively.^7^ Even with a relatively high vaccination coverage, widespread community transmission appeared in late March and peaked in April. A series of strict non‐pharmaceutical interventions (NPIs) were implemented to contain the outbreak, such as case isolation, contact tracing, mass testing, and city‐wide lockdown.^8^ As of May 31, 2022, when the lockdown was lifted, over 0.6 million confirmed cases including 588 deaths were reported in Shanghai.^9^

Transmission dynamics of SARS‐CoV‐2 may potentially evolve over time and vary by settings and with intervention measures. Households are important transmission venues for SARS‐CoV‐2.^10^, ^11^, ^12^ A full understanding of the household transmission patterns of SARS‐CoV‐2 Omicron variant is crucial to plan and adjust the public health responses and target intervention in face of the current challenge of Omicron epidemics. Recently, a few studies from Denmark, Norway, and the United States have reported higher household secondary attack rates (25.1%–52.7%) for Omicron variant than for Delta variant.^13^, ^14^, ^15^, ^16^, ^17^ However, accurately determining the household transmission dynamics regardless of symptoms remains challenging, as most studies were based on the analysis of symptom‐based screening data, with asymptomatic infections and mild non‐medically consulted infections underreported. This challenge can be addressed by studies of close contacts with routine SARS‐CoV‐2 testing regardless of symptoms to detect asymptomatic and mildly symptomatic cases. As household contacts of SARS‐CoV‐2‐positive cases are likely to be highly exposed to the case and are known to be at high risk of infection, they are an ideal group shedding lights on SARS‐CoV‐2 transmission dynamics.^18^

Here, we conducted a case‐ascertained study to determine the features of household transmission of SARS‐CoV‐2 Omicron variant in Shanghai, China. In particular, we estimated the distribution of key time‐to‐event intervals, quantified the household transmission risk and explored the transmission heterogeneities in infectivity and susceptibility. In the meantime, we also assessed the vaccine effectiveness of inactivated COVID‐19 vaccines against Omicron infection and symptomatic disease.

## 2 METHODS

### 2.1 Study design and participants

**FIGURE 1:**
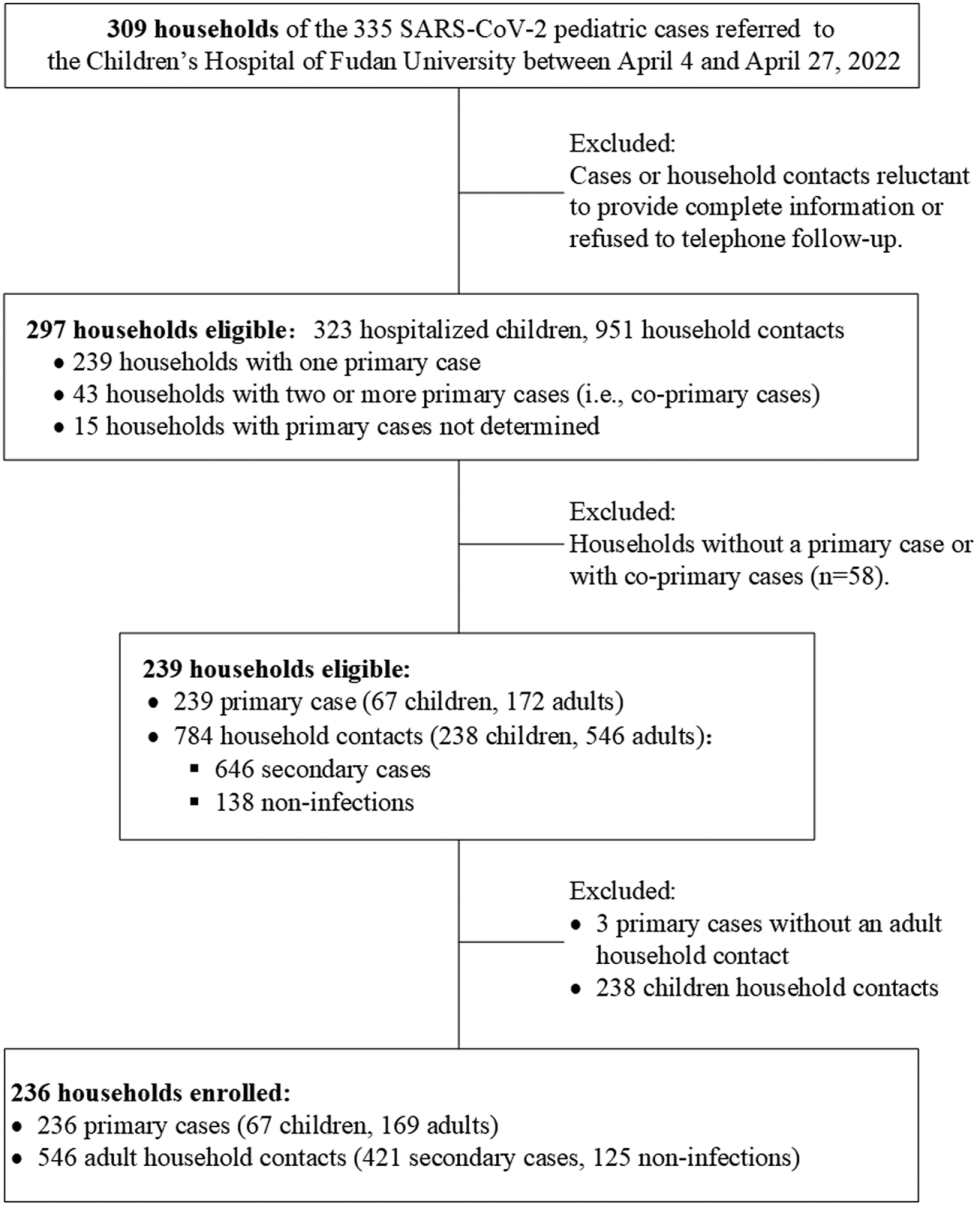
Flow chart describing the procedure for screening study participants

Between April 4 to April 27, 2022, a total of 335 SARS‐CoV‐2 pediatric cases from the 309 households were referred to the Children's Hospital of Fudan University, a designated hospital for management of pediatric COVID‐19 cases in Shanghai (Figure 1). All these cases were laboratory‐confirmed before hospitalization, with suspected pneumonia or comorbidities requiring special medical attention. During the outbreak, asymptomatic and milder pediatric cases were usually transferred to designated isolation facilities for medical observation. Each pediatric case was allowed to have their parents accompanying during hospital stay. Routine medical observations and PCR testing for the hospitalized children and their accompanying parents were conducted at the hospital. Other family member contacts were mandatorily required for 14‐day isolation and quarantine at the community isolation facilities or centers and received PCR screening for SARS‐CoV‐2 every 2 days and even every day. If they developed any symptom or sign of COVID‐19, an additional test was done to help timely detect infection. Cases with two consecutive RT‐PCR (reverse transcription‐polymerase chain reaction) negative testing results (i.e., the Cycle threshold value for SARS‐CoV‐2, Ct ≥ 35) were discharged from isolation.^19^

We conducted a case‐ascertained study to capture the information of all these 309 households of the 335 pediatric cases, including the demographics, exposures, vaccination status, infection and clinical information of both the hospitalized children and their household members (Table S1). In‐depth household investigations were conducted during the hospital stay (face‐to‐face interview with the accompanying parents using a standard questionnaire, Table S1) and after one‐week of discharge (routine telephone follow‐up, to check the infection status of each household member). The households were excluded from the study if any of the cases or household members were reluctant to provide the complete information or refused to telephone follow‐up. Complete information was collected from the 297 households, including 323 hospitalized children and their 951 household members (Figure 1).

Based on the detailed information obtained from household investigations, we defined the primary case for each household as a confirmed case with a history of community exposure (i.e., exposed to SARS‐CoV‐2 contaminated environment or contact with a confirmed case in the community). For a household without determined source of infection, we defined the primary case as the first individual who was tested positive with RT‐PCR or developed symptoms. Other household members with positive RT‐PCR results were defined as secondary cases. To reduce the potential uncertainty of the results, we only focused on those households with a single primary case. After excluding households without a primary case or with co‐primary cases, 239 households were eligible, including 239 primary cases and their 784 household contacts, among which 646 secondary cases were identified (Figure 1).

In this study, all the enrolled households came from the hospitalized pediatric cases of COVID‐19 who was either a primary or a secondary case. To avoid overestimation of the household secondary attack rates due to the selection bias of household enrollment, the secondary infection and clinical attack rates (SARI and SARC), as well as the transmission heterogeneities and vaccine effectiveness (VE) measured based on SARI and SARC, were estimated among adult household contacts. For this reason, we further excluded three households without an adult household contact from the 239 enrolled households, and finally, a total of 236 households were qualified for analysis, including 236 primary cases and 546 adult household contacts (Figure 1).

### 2.2 Case definition and household contact

In this study, a confirmed case is defined as a person with PCR‐confirmed SARS‐CoV‐2 infection, irrespective of clinical signs and symptoms. A symptomatic case is defined as confirmed cases who develop COVID‐19‐related symptoms, such as fever, cough, runny nose, sore throat, diarrhea, vomit and constitutional symptoms, and further classified as mild, moderate (non‐severe pneumonia), severe and critical case based on both the national and World Health Organization (WHO) guidance,^19^, ^20^ otherwise, they will be defined as asymptomatic cases. Pneumonia was diagnosed based on either radiological evidence or typical clinical signs (fever and or cough accompanying with one of the following signs: moist rales, difficulty in breathing, fast breathing, chest indrawing). A household is defined as two or more people living in the same residence. A household contact is defined as any person who has resided in the same household with a confirmed case for the period from 2 days before to 14 days after the date of symptom onset or laboratory confirmation. Each hospitalized pediatric case in this study had at least one household contacts.

### 2.3 Statistical analysis

We estimated the incubation period (i.e., the period of time from an exposure resulting in SARS‐CoV‐2 infection to symptom onset) by analyzing cases with clear exposure history. When cases reported multiple or sustained exposures, any time within their exposure windows was considered to be their possible infection time. We also estimated the serial interval (i.e., the time interval between the onset of symptoms in a primary case and his/her secondary cases), as well as the infectiousness profile (i.e., the distribution of the time interval from the onset of symptoms in a primary case to the infection in his/her secondary cases). For a secondary case contacts with multiple infections, we randomly selected one as his/her primary case and simulated 100 times to account for potential uncertainties (see Sun. et al for more details).^21^ A sensitivity analysis for the situation that all secondary cases are from the same primary cases was also conducted. We fitted three parametric distributions (Weibull, gamma, and lognormal) to time‐to‐event data and selected the best fit based on the minimum Akaike information criterion. The distributions of serial interval and the infectiousness profile were fitted with a shift parameter allowing negative values.

We further excluded the households without a primary case or with co‐primary cases from the analysis to avoid potential bias, as it is possible that a secondary case may be misclassified as a co‐primary case (Figure 1). The secondary infection attack rate (SAR_I_) was defined as the number of PCR‐confirmed cases detected regardless of symptom among all household contacts of the primary case.^18^ The secondary clinical attack rate (SAR_C_) was defined as the number of symptomatic cases detected among all household contacts of the primary case.^18^ In this study, there was a potential bias in the estimates of SAR_I_ and SAR_C_ among children household contacts due to the study design that the enrolled households were selected from the families of the confirmed hospitalized pediatric cases. Therefore, we estimated the SAR_I_ and SAR_C_ among adult household contacts to assess the heterogeneities in infectivity and susceptibility. Specifically, the heterogeneities in susceptibility were estimated by the characteristics (e.g., sex and vaccination status) of adult household contacts. The heterogeneities in infectivity were measured by the characteristics (e.g., age, sex, household size, symptom profile, and vaccination status) of primary cases (including children and adults).

Comparison between groups was performed using chi‐square test. A difference with *P* < 0.05 at two‐side was considered to be statistically significant. We estimated the vaccine effectiveness against Omicron infection (VE_I_) and against clinical symptoms (VE_C_) based on the estimates of SAR_I_ and SAR_C_ among adult household contacts with different vaccination status. Specifically, the estimates of VE_I_ were obtained from ${\mathrm{VE}}_{I,v}=1-\left({\mathrm{SAR}}_{I,v}/{\mathrm{SAR}}_{I,u}\right)$, where v=1,2,3, donates the partially, fully and booster vaccinated groups among the adult household contacts, respectively. ${\mathrm{SAR}}_{I,v}$ donates the secondary infection rate of each vaccinated group and ${\mathrm{SAR}}_{I,u}$ donates that of the unvaccinated group. Similarly, the estimates of VE_C_ were obtained from ${\mathrm{VE}}_{C,v}=1-\left({\mathrm{SAR}}_{C,v}/{\mathrm{SAR}}_{C,u}\right)$, where ${\mathrm{SAR}}_{C,v}$ donates the secondary clinical attack rate of each vaccinated group and ${\mathrm{SAR}}_{C,u}$ denotes that of the unvaccinated group. Statistical analysis was preformed using the R software, version 4.0.2; the data were stored and maintained using Microsoft Office Excel 2019.

## 3 RESULTS

A total of 297 households, including 323 hospitalized pediatric cases and their 951 household members, were initially recruited to the study (Figure 1). The epidemiological and clinical characteristics were described in Table S2 and Figure S1. All household cases in this study were non‐severe or asymptomatic, except a 7‐year‐old child, who was critically ill. The mean time interval between first enrollment and last follow‐up was 20.9 days for each household.

**FIGURE 2:**
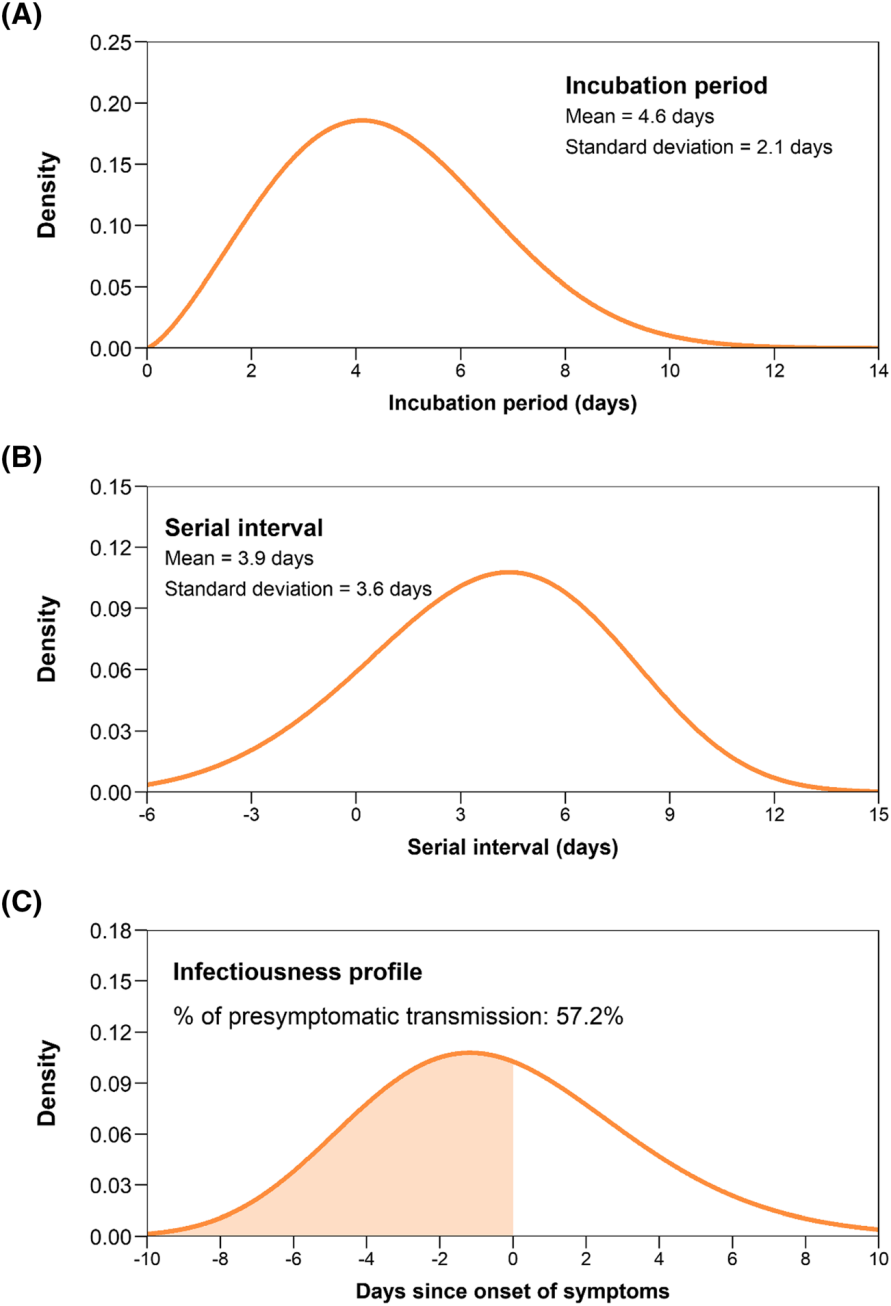
Best fitted distributions of the incubation period and serial interval and infectiousness profile since onset of symptoms. (A). Estimated distribution of the incubation period based on the analysis of 52 confirmed cases from 28 households. (B). Estimated distribution of the serial interval based on the analysis of 234 transmission pairs. (C) Estimated infectiousness profile since onset of symptoms based on the analysis of 234 transmission pairs

We analyzed the period of time from exposure resulting in SARS‐CoV‐2 infection to disease onset for the 52 symptomatic cases with clear exposure history. We estimated a mean incubation period of 4.6 (median: 4.4, IQR: 3.1–6.0) days, with a standard deviation (sd) of 2.1 days and the 95th percentile of the distribution at 8.3 days (Figure 2A). The incubation period was well approximated by a Weibull distribution (Table S3). We estimated the distribution of serial interval and the infectiousness profile based on the 234 transmission pairs (accounting for the uncertainty caused by co‐primary cases). The serial interval followed a best fitted Weibull distribution with an estimated mean of 3.9 (median: 4.0, COVID‐19‐related symptoms IQR 1.4–6.4) days and a standard deviation of 3.6 days (Figure 2B and Table S4), while the infectiousness profile followed a gamma distribution with 57.2% of the transmission events occurred at the presymptomatic phase (Figure 2C and Table S5). Sensitivity analysis (for the situation that all secondary cases of a household are from the same primary case, not accounting for the uncertainty caused by co‐primary cases) showed similar results, with an estimated serial interval of 3.8 ± 3.8 days and 53.3% of the household transmission occurred at the presymptomatic phase (Figure S2).

Then, we excluded 43 households with co‐primary cases, 15 households without primary cases determined and three households without adult household contacts (see Method section for details). We finally included 236 primary cases and their 546 adult household contacts for analysis (Figure 1). The characteristics of the 236 primary cases and their 546 adult household contacts were described in Table 1. Among the 236 household primary cases, 169 (71.61%) were adults and 134 (56.78%) were females. Only 89 (37.71%) of the primary cases reported a clear history of community SARS‐CoV‐2 exposure, indicating most of the households without a determined source of infection. We found 37.71% of the primary cases were unvaccinated, 2.97%, 35.59%, and 23.73% of the primary cases received partial, full and booster vaccination, respectively. Most primary cases (89.83%) were symptomatic. Among the 546 adult household contacts, 421 secondary cases were identified and 366 (86.94%) developed symptoms. The overall SAR_I_ and SAR_C_ among adult household contacts were 77.11% (95% CI: 73.58%–80.63%) and 67.03% (95% CI: 63.09%–70.98%), respectively. The heterogeneity in terms of SAR_I_ and SAR_C_ across households were shown in Figure S3, indicating that in 64.83% (153/236) of the households, all adult household contacts were finally infected and in 54.24% (128/236) of the households, all adult household contacts developed symptoms. We found the infectivity was not significantly different in primary cases with different sex, vaccination status, and household size. There was also no significant difference in infectivity between child and adult infections, as well as symptomatic and asymptomatic cases (*P* > 0.05, Table 2). For the transmission heterogeneities in susceptibility, we found a higher proportion of females (59.86% vs. 38.4%) and a lower proportion of vaccinated individuals (76.72% vs 89.9%) in secondary cases than in uninfected household contacts (Table 1). Accordingly, we found higher susceptibility to SARS‐CoV‐2 Omicron infection in females (SAR_I_ = 84%) within household than in males (SAR_I_ = 68.7%, *P* < 0.001). Similar conclusion was reached when the susceptibility was measured by SAR_C_ (74% for females and 58.54% for males, *P* < 0.001). Unvaccinated adults were associated with the highest risk of household infection (SAR_I_ = 88.29%) and symptomatic infection (SAR_c_ = 81.08%), while SAR_I_ could be reduced to 84%, 75.21% and 71.59% (*P* = 0.007), and SAR_C_ could be reduced to 76%, 63.68%, and 61.36% (*P* = 0.002), through partial, full, and booster vaccination, respectively (Table 3).

**TABLE 1:** Characteristics of the 236 primary cases and their 546 adult household contacts

Characteristics	Primary cases (*N* = 236)	Adult household contacts	
		Secondary cases (*N* = 421)	Uninfected contacts (*N* = 125)
**Age group, years**			
0–17	67 (28.39)	0 (0)	0 (0)
18+	169 (71.61)	421 (100)	125(100)
**Sex**			
Male	102 (43.22)	169 (40.14)	77 (61.6)
Female	134 (56.78)	252 (59.86)	48 (38.4)
**Community exposure**			
Yes	89 (37.71)	0 (0)	0 (0)
No	0 (0)	421 (100)	125(100)
Not determined	147 (62.29)	0 (0)	0 (0)
**Vaccination status** ^a^			
Unvaccinated	89 (37.71)	98 (23.28)	13 (10.4)
Partial	7 (2.97)	21 (4.99)	4 (3.2)
Full	84 (35.59)	176 (41.81)	58 (46.4)
Booster	56 (23.73)	126 (29.93)	50 (40)
**Symptom status**			
Symptomatic	212 (89.83)	366 (86.94)	‐
Asymptomatic	24 (10.17)	55 (13.06)	‐

^a^ Partial vaccination was defined as an individual receiving only one‐dose inactivated vaccine. Full vaccination was defined as an individual receiving two doses of inactivated SARS‐CoV‐2 vaccines for at least 2 weeks. Booster vaccination was defined as a fully vaccinated individual receiving an additional dose of inactivated vaccine for at least 14 days.

**TABLE 2:** Infectivity of primary cases, measured by secondary infection attack rate (SAR_I_) and secondary clinical attack rate (SAR_C_), based on the analysis of 236 primary cases and their 546 adult household contacts^a^

Characteristics of primary cases	No. of primary cases (*N* = 236)	No. of adult household contacts (*N* = 546)	No. of secondary cases (*N* = 421)	Infectivity measured by SAR_I_, % (95% CI)	*P* value	No. of secondary cases developing symptoms (*N* = 366)	Infectivity measured by SAR_C_, % (95% CI)	*P* value
**Age group, years**								
0–17	67	175	129	73.71 (67.19–80.24)	0.235	112	64 (56.89–71.11)	0.348
18+	169	371	292	78.71 (74.54–82.87)		254	68.46 (63.74–73.19)	
**Sex**								
Male	102	240	193	80.42 (75.4–85.44)	0.127	168	70 (64.2–75.8)	0.225
Female	134	306	228	74.51 (69.63–79.39)		198	64.71 (59.35–70.06)	
**Vaccination status**								
Unvaccinated	89	221	162	73.3 (67.47–79.14)	0.066	140	63.35 (57–69.7)	0.240
Partial^b^	7	12	7	‐		6	‐	
Full	84	187	146	78.07 (72.14–84)		131	70.05 (63.49–76.62)	
Booster	56	126	106	84.13 (77.75–90.51)		89	70.63 (62.68–78.59)	
**Clinical severity**								
Symptomatic	212	480	373	77.71 (73.98–81.43)	0.455	329	68.54 (64.39–72.7)	0.060
Asymptomatic	24	66	48	72.73 (61.98–83.47)		37	56.06 (44.09–68.03)	
**Household size**								
2–3	66	85	69	81.18 (72.87–89.49)	0.406	64	75.29 (66.13–84.46)	0.102
4–9	170	461	352	76.36 (72.48–80.23)		302	65.51 (61.17–69.85)	

^a^ Households without a primary case or with co‐primary cases were excluded from this analysis. We assessed the infectivity of primary cases among their adult household contacts to avoid potential bias due to the study design (detailed in Section 2).

^b^ The SAR_I_ and SAR_C_ among adult household contacts of a partially vaccinated primary case were not estimated due to the extremely small sample size (i.e., 12 household contacts corresponding to seven primary cases).

**TABLE 3:** Susceptibility of adult household contacts, measured by secondary infection attack rate (SAR_I_) and secondary clinical attack rate (SAR_C_), based on the analysis of 546 adult household contacts from 236 households^a^

Characteristics of adult household contacts	No. of adult household contacts	No. of secondary cases	Susceptibility measured by SAR_I_, % (95% CI)	*P* value	No. of secondary cases developing symptoms	Susceptibility measured by SAR_C_, % (95% CI)	*P* value
Overall	546	421	77.11 (73.58–80.63)	‐	366	67.03 (63.09–70.98)	‐
**Sex**							
Male	246	169	68.7 (62.9–74.49)	<0.001	144	58.54 (52.38–64.69)	<0.001
Female	300	252	84 (79.85–88.15)		222	74 (69.04–78.96)	
**Vaccination status**							
Unvaccinated	111	98	88.29 (82.31–94.27)	0.007	90	81.08 (73.79–88.37)	0.002
Partial	25	21	84 (69.63–98.37)		19	76 (59.26–92.74)	
Full	234	176	75.21 (69.68–80.75)		149	63.68 (57.51–69.84)	
Booster	176	126	71.59 (64.93–78.25)		108	61.36 (54.17–68.56)	

^a^ Households without a primary case or with co‐primary cases were excluded from this analysis. We assessed the susceptibility among adult household contacts to avoid potential bias due to the study design (detailed in Section 2).

Full vaccination was 14.8% (95% CI: 5.8%–22.9%) and 21.5% (95% CI: 10.4%–31.2%) effective against Omicron infection and symptomatic disease. The estimated VE of booster vaccination was 18.9% (95% CI: 9.0%–27.7%) against Omicron infection and 24.3% (95% CI: 12.3%–34.7%) against symptomatic disease. By contrast, partial vaccination has no significant effect on preventing Omicron infection (4.9%, 95%CI: −14.4%‐20.8%) and symptomatic disease (6.3%, 95%CI: −18.9%–26.1%) (Table 4).

**TABLE 4:** Effectiveness of inactivated vaccines (VE) against SARS‐CoV‐2 Omicron infection and symptomatic disease

Vaccination status	VE against infection	VE against symptomatic infection
Unvaccinated	Ref	Ref
Partial	4.9 (−14.4, 20.8)	6.3 (−18.9, 26.1)
Full	14.8 (5.8, 22.9)	21.5 (10.4, 31.2)
Booster	18.9 (9.0, 27.7)	24.3 (12.3, 34.7)

## 4 DISCUSSION

This study of household transmission patterns is based on a well‐designed case‐ascertained study during the Omicron wave in Shanghai, China, with detailed household investigations and consecutively intensive RT‐PCR testing. Our results showed high risk of household transmission due to the transmission from pre‐symptomatic and asymptomatic infections, despite the implementation of city‐wide lockdown and centralized isolation/quarantine of cases and close contacts in hospitals or designated facilities. We observed no significant difference in transmissibility between child and adult infections and symptomatic and asymptomatic individuals, while the susceptibility to Omicron infection among female household contacts was higher than males. Our findings also implied marginal effectiveness of inactivated vaccines against Omicron infection and symptomatic diseases, although inactivated vaccines may show high effectiveness against severe outcomes.^19^, ^20^, ^22^

In this study, detailed information on exposures and symptoms of the study participants was collected through in‐depth household investigations, allowing us to provide robust estimation of several key time‐to‐event distributions. We observed a mean incubation period of 4.6 ± 2.1 days for Omicron variant, slightly longer than prior estimates for Omicron (3.0–3.6 days)^23^, ^24^, ^25^, ^26^, ^27^ while shorter than that of the ancestral strain (6.3 days).^28^ The 95th percentile of the incubation period distribution was at 8.3 days, suggesting the feasibility of a shorter quarantine period for close contacts or population at risk. Additionally, studies from Spanish, Netherlands, South Korea, Belgium, and the United States showed shorter serial intervals for Omicron, with the mean estimates ranging from 2.75–4.8 days.^13^, ^27^, ^29^, ^30^, ^31^, ^32^, ^33^ In agreement with prior findings, we observed a mean serial interval of 3.9 ± 3.7 days, falling within this interval. Shortened serial intervals suggested increased transmissibility and growth advantage of Omicron variant, making timely contact tracing more challenging.^34^ The proportion of the presymptomatic transmission was estimated at 57.2%. However, it's important to stress that our estimates account for the possible effect of NPIs, especially case isolation and contact tracing, which truncate the transmission chains within household, leaving most of the transmission events occurs at the early phase of infection. Similar patterns have been reported in previous studies in terms of SARS‐CoV‐2 ancestral strains and Delta variant.^35^, ^36^

Omicron infection resulted in high attack rates among household contacts in this investigation. Although the precise age of each participant was not collected, it's important to note that the adult household contacts in this study should be a relatively young population, with 63.7% (348/546) of them were parents of the pediatric cases, 31.5% (172/546) were grandparents of the pediatric cases, and 4.8% (26/546) were other household contacts living together with the pediatric cases, such as old brothers/sisters, uncles/aunts, and babysitters. On the other hand, the enrolled pediatric cases were almost younger children, thus, the grandparents were not very old. We estimated the overall SAR_I_ among adult household contacts to be 77.11%, around 2.5–6 times higher than previous estimates (13.2%–31.6%) in Wuhan, Zhejiang, Shenzhen, Guangzhou, and Beijing during the first COVID‐19 wave in China when the national lockdown was implemented,^11^, ^37^, ^38^, ^39^, ^40^ consistent with prior studies indicating increased transmissibility of Omicron to preexisting variants.^14^, ^16^, ^41^ The overall estimates of SAR_C_ among adult household contacts in our study were 67.03%, higher than that reported in the US (52.7%), Denmark (31%), and Norway (25.1%).^16^, ^17^, ^18^ This may be partially explained by the longer duration and higher frequency of contacts between household members during the lockdown period, as well as the circulation of more transmissible and immune evasive Omicron BA.2 sublineage.^17^ Additionally, the extremely low level of immunity against SARS‐CoV‐2 induced by natural infection among population in Shanghai was also directly correlated with high household attack rates. Of particular note, our investigations almost capture all household secondary infections as centralized quarantine and intensive RT‐PCR testing were mandated for all household contacts regardless of symptom during the period of outbreak. Besides, despite strict NPIs were implemented in Shanghai (e.g., city‐wide lockdown, stay‐at‐home order, mass testing, and isolation/quarantine of all SARS‐CoV‐2 infections and close contacts), our study showed that transmission from pre‐symptomatic and asymptomatic infections largely reduced the impact of interventions on stopping the household transmission, stressing the importance of early detection and timely isolation of the confirmed cases and quarantine of their contacts.

During the Omicron wave, substantial increase in pediatric cases of COVID‐19 was reported in the United States.^42^ However, the role of children in Omicron transmission has yet to be fully understood. We observed similar high infectivity in pediatric cases (aged 0–17 years) and in adults (aged 18+ years), indicating that children played an equal role in Omicron transmission in household as adults. Our finding also demonstrated the similar high‐level transmission rate from symptomatic and asymptomatic primary cases, which implies that symptom‐based surveillance is insufficient to prevent and control of COVID‐19 epidemic, posing great challenge for prevention and control of Omicron transmission. We found females were more susceptible to Omicron infection in household than males, in line with the finding reported in an early study from Wuhan.^11^ Part explanation was that females are more likely to take care of the sick individuals, involve more housework in household and accompany sick children at the hospital. Of particular note, we observed significantly higher susceptibility to Omicron infection for unvaccinated household contacts, consistent with the findings reported in the latest studies from the US, Denmark and Norway.^13^, ^14^, ^15^, ^16^, ^41^ The estimated VEs against Omicron infection and symptomatic disease was 14.8% and 21.5% for fully vaccination, and 18.9% and 24.3% for booster vaccination. An updated meta‐analysis based on four household transmission studies from Denmark, Norway, and the Unites States reported that the effectiveness of mRNA vaccines for fully vaccinated contacts was 18.1%,^41^ which is similar to our findings. The marginal VEs against Omicron infection and mild disease suggest significant immune escape of Omicron variant to vaccine‐induced antibody protection and waning vaccine immunity over time.^43^, ^44^ However, the role of the current COVID‐19 vaccines remains valuable in minimizing the direct disease burden of SARS‐CoV‐2 Omicron variant because VE estimates against the Omicron variant remain higher for severe disease in the majority of studies.^44^ For severe disease caused by Omicron variant, VE of the primary series showed little decline over 6 months and the first booster dose vaccination improved VE (≥70%) following three to 6 months from a booster dose.^44^

Household transmission patterns are somewhat heterogeneous across studies. The accuracy of the results may be affected by a high degree of methodologic heterogeneity with respect to method and frequency of testing for diagnosis of contacts, isolation of cases and duration of follow‐up. A major strength of this study is that we captured more secondary symptomatic and asymptomatic infections of the recruited households as all household members received consecutive RT‐PCR testing for SARS‐CoV‐2 after a primary household case was identified. The estimation of VE is more objective because exposure risk and contact pattern of household individuals are equal and homogeneous relative to the population‐based observational study. However, our study is not without limitations. First, despite in‐depth household investigation and follow‐up of each case, we could not always reconstruct the entire transmission chain and fully avoid recall bias in individual records. We tried to collect information on source of exposures for each household to avoid potential bias, but there are still some households without determined source of infection. The primary cases of these households were defined as the first household members with positive RT‐PCR testing results or the sign of COVID‐19 symptoms, which may misclassify the primary and secondary cases of households. Second, due to the study design, there was at least one pediatric case in each enrolled household. We can only estimate the transmission risk among adult household contacts, Further studies are needed to assess the susceptibility to Omicron infections among pediatric household contacts. Moreover, for those households with an adult primary case, close contact usually inevitable when caring for secondary pediatric cases, which might significantly increase the risk of COVID‐19 infection to the caregiver and could possibly lead to an overestimation of the adult household attack rate. Third, we did not collect specific age of household contacts, only classified them as children (i.e., 0–17 years) and adults (i.e., 18+ years). Although we concluded that there was no significant difference in infectivity between child and adult infections, the age‐specific infectivity needs to be further explored. Finally, due to the lack of precise age information on all adult household members and limited sample size of uninfected participants (e.g., only 13 unvaccinated and our partially vaccinated adult household contacts remained uninfected, Table 1), we only provide VE estimates based on univariate analysis. Further studies with detailed age information and large sample size should be conducted and provide estimates corrected for multiple factors.

In conclusion, high household transmission during the Omicron wave in Shanghai indicates the importance of early detection and timely isolation of SARS‐CoV‐2 infections. Marginal effectiveness of inactivated vaccines against Omicron infection poses a great challenge for the prevention and control of the SARS‐CoV‐2 Omicron variant, implying the necessity of optimizing vaccine strategies.

## CONFLICT OF INTEREST

All the authors declared no conflicts of interest related to this work.

## ETHICS STATEMENT

This study was approved by the Ethics Committee of the Children's Hospital of Fudan University [Ethics Ref: NO. (2021)29].

## AUTHOR CONTRIBUTIONS

**Zhongqiu Wei:** Conceptualization; data curation; formal analysis; investigation; methodology; software; validation; visualization; writing‐original draft. **Wenjie Ma:** Data curation; formal analysis; methodology; software; validation; visualization; writing‐original draft. **Zhonglin Wang:** Data curation; investigation; methodology; validation; visualization; writing‐original draft. **Jingjing Li:** Data curation; formal analysis; investigation; methodology; software. **Xiaomin Fu:** Data curation; formal analysis; investigation; methodology. **Hailing Chang:** Data curation; investigation. **Yue Qiu:** Data curation; investigation. **He Tian:** Data curation; investigation; resources. **Yanfeng Zhu:** Data curation; methodology. **Aimei Xia:** Investigation; methodology; resources. **Qianhui Wu:** Formal analysis; methodology; software. **Gongbao Liu:** Methodology; project administration; resources. **Xiaowen Zhai:** Methodology; project administration; resources; supervision. **Xiaobo Zhang:** Methodology; project administration; resources. **Yan Wang:** Conceptualization; formal analysis; methodology; validation; visualization; writing‐review and editing. **Mei Zeng:** Conceptualization; funding acquisition; methodology; project administration; supervision; validation; visualization; writing‐review and editing.

### PEER REVIEW

The peer review history for this article is available at https://publons.com/publon/10.1111/irv.13097.

## Supporting information


**Table S1.** Questionnaire.
**Table S2.** Epidemiological and clinical characteristics of the 323 pediatric cases and their 951 household members.
**Table S3.** Estimates of the incubation period based on the analysis of 52 cases from 28 households.
**Table S4.** Estimates of the serial interval based on the analysis of 234 transmission pairs.
**Table S5.** Estimates of the infectiousness profile based on the analysis of 234 transmission pairs.
**Figure S1.** Vaccination status of the 323 hospitalized pediatric cases and their 951 household members.
**Figure S2.** Best fitted distributions of the serial interval and infectiousness profile since onset of symptoms (sensitivity analysis).
**Figure S3.** The heterogeneity in terms of secondary infection attack rates and secondary clinical attack rates across households.

## Data Availability

The data and codes that support the findings of this study are available from the corresponding author upon reasonable request.
