# Supplementary material for: Household transmission of SARS‐CoV‐2 during the Omicron wave in Shanghai, China: A case‐ascertained study
Source: Influenza Other Respir Viruses. 2023 Feb 5;17(2):e13097. doi: 10.1111/irv.13097 (PMC9946695; doi:10.1111/irv.13097)
Supplement: Supplementary file 1 — Table S1. Questionnaire. Table S2. Epidemiological and clinical characteristics of the 323 pediatric cases and their 951 household members. Table S3. Estimates of the incubation period based on the analysis of 52 cases from 28 households. Table S4. Estimates of the serial interval based on the analysis of 234 transmission pairs. Table S5. Estimates of the infectiousness profile based on the analysis of 234 transmission pairs. Figure S1. Vaccination status of the 323 hospitalized pediatric cases and their 951 household members. Figure S2. Best fitted distributions of the serial interval and infectiousness profile since onset of symptoms (sensitivity analysis). Figure S3. The heterogeneity in terms of secondary infection attack rates and secondary clinical attack rates across households. [file IRV-17-e13097-s001.docx]

Supplementary Materials

for

**Household transmission of SARS-CoV-2 during the Omicron wave in Shanghai, China: a case-ascertained study**

**Table of contents**

**Supplementary Tables:**

Supplementary Table 1. Questionnaire.

Supplementary Table 2. Epidemiological and clinical characteristics of the 323 pediatric cases and their 951 household members.

Supplementary Table 3. Estimates of the incubation period based on the analysis of 52 cases from 28 households.

Supplementary Table 4. Estimates of the serial interval based on the analysis of 234 transmission pairs.

Supplementary Table 5. Estimates of the infectiousness profile based on the analysis of 234 transmission pairs.

**Supplementary Figures**

Supplementary Figure 1. Vaccination status of the 323 hospitalized pediatric cases and their 951 household members.

Supplementary Figure 2. Best fitted distributions of the serial interval and infectiousness profile since onset of symptoms (sensitivity analysis).

Supplementary Figure 3. The heterogeneity in terms of secondary infection attack rates and secondary clinical attack rates across households.

**Supplementary Table 1.** Questionnaire.

| **Case initial reporting form - for hospitalized pediatric cases** | |
| --- | --- |
| **Basic information** |  |
| Case ID |  |
| Case name |  |
| Age (years, months) | years months  Unknown |
| Sex | Male  Female |
| Household size |  |
| Telephone (mobile) number |  |
| Date of first interview (dd/mm/yyyy) | / / |
| Date of follow up (dd/mm/yyyy) | / / |
| **Exposure history** |  |
| Exposure place | Community exposure  Household exposure  Other places: |
| Exposure start date (dd/mm/yyyy) | / / |
| Exposure end date (dd/mm/yyyy) | / / |
| **Vaccination status** |  |
| Vaccination history | Yes  No |
| Manufacture of the vaccines |  |
| Doses of vaccination | Unvaccinated  Partial  Full  Booster |
| Date of the last vaccination (dd/mm/yyyy) | / /  Unknown |
| **Clinical information** |  |
| Date of first positive result of nucleic acid testing or antigen testing (dd/mm/yyyy) | / / |
| Clinical severity | Asymptomatic  Mild  Moderate  Severe  Critical |
| Date of symptom onset (dd/mm/yyyy) | / /  Asymptomatic infection  Unknown |
| Date of admission (dd/mm/yyyy) | / / |
| Date of discharge (dd/mm/yyyy) | / / |
| **Symptoms:** |  |
| Fever (≥37.5°C) or history of fever | Yes  No  Unknown  If Yes, please specify fever spike: °C and fever duration: days |
| Cough | Yes  No  Unknown |
| Nasal obstruction | Yes  No  Unknown |
| Nausea | Yes  No  Unknown |
| Vomiting | Yes  No  Unknown |
| Diarrhoea | Yes  No  Unknown |
| Loss of smell (anosmia) | Yes  No  Unknown |
| Loss of taste | Yes  No  Unknown |
| Muscle aches | Yes  No  Unknown |
| **Underlying disease** |  |
| pre-existing condition/co-morbidity | Yes  No  Unknown  If Yes, please specify: |
| **Imagology and laboratory testing** |  |
| X-ray or CT image characteristics |  |
| White blood cell count (*10^9/L) |  |
| Neutrophil count (*10^9/L) |  |
| Lymphocyte count (*10^9/L) |  |
| Platelet count (*10^9/L) |  |
| C-reactive protein (mg/L) |  |
| **Case initial reporting form - for household members of the pediatric cases** | |
| **Basic information** |  |
| Household Contact ID |  |
| Related hospitalized children |  |
| Relationship with the hospitalized children |  |
| Age | 0-17  18+ |
| Sex | Male  Female |
| Telephone (mobile) number |  |
| Date of first interview (dd/mm/yyyy) | / / |
| Date of follow up (dd/mm/yyyy) | / / |
| **Exposure history** |  |
| Exposure place | Community exposure  Household exposure  Other places: |
| Exposure start date (dd/mm/yyyy) | / / |
| Exposure end date (dd/mm/yyyy) | / / |
| **Vaccination status** |  |
| Vaccination history | Yes  No |
| Manufacture of the vaccines |  |
| Doses of vaccination | Unvaccinated  Partial  Full  Booster |
| Date of the last vaccination (dd/mm/yyyy) | / /  Unknown |
| **Clinical information** |  |
| PCR confirmed cases? | Yes  No |
| Date of first positive result of nucleic acid testing or antigen testing (dd/mm/yyyy) | / / |
| Symptomatic cases? | Yes  No |
| Date of symptom onset (dd/mm/yyyy) | / /  Asymptomatic infection  Unknown |
| **Symptoms:** |  |
| Fever (≥37.5°C) or history of fever | Yes  No  Unknown |
| Cough | Yes  No  Unknown |
| Nasal obstruction | Yes  No  Unknown |
| Nausea | Yes  No  Unknown |
| Vomiting | Yes  No  Unknown |
| Diarrhoea | Yes  No  Unknown |
| Loss of smell (anosmia) | Yes  No  Unknown |
| Loss of taste | Yes  No  Unknown |
| Muscle aches | Yes  No  Unknown |

**Supplementary Table 2.** Epidemiological and clinical characteristics of the 323 pediatric cases and their 951 household members.

| **Characteristics** | **Hospitalized pediatric cases** | | | **Household members** | |
| --- | --- | --- | --- | --- | --- |
|  | **Non-pneumonia cases**  **(N=279)** | **Pneumonia cases**  **(N=44)** | **Overall**  **(N=323)** | **Infected**  **(N=787)** | **Uninfected**  **(N=164)** |
| **Age group, years** | n (%) | n (%) | n (%) | n (%) | n (%) |
| Median (IQR) | 2 (0.83-4.08) | 2 (0.98-3.5) | 2 (0.83-4) | - | - |
| **0-17** | **279 (100)** | **44 (100)** | **323 (100)** | **32 (4.07)** | **15 (9.15)** |
| 0-1 | 79 (28.32) | 11 (25) | 90 (27.86) | - | - |
| 1-2 | 97 (34.77) | 15 (34.09) | 112 (34.67) | - | - |
| 3-5 | 48 (17.2) | 14 (31.82) | 62 (19.2) | - | - |
| 6-12 | 44 (15.77) | 3 (6.82) | 47 (14.55) | - | - |
| 13-17 | 11 (3.94) | 1 (2.27) | 12 (3.72) | - | - |
| **18+** | **0 (0)** | **0 (0)** | **0 (0)** | **755 (95.93)** | **149 (90.85)** |
| **Sex** |  |  |  |  |  |
| Male | 168 (60.22) | 26 (59.09) | 194 (60.06) | 313 (39.77) | 101 (61.59) |
| Female | 111 (39.78) | 18 (40.91) | 129 (39.94) | 474 (60.23) | 63 (38.41) |
| **Community exposure** |  |  |  |  |  |
| Yes | 22 (7.89) | 5 (11.36) | 27 (8.36) | 101 (12.83) | 0 (0) |
| No | 190 (68.1) | 32 (72.73) | 222 (68.73) | 501 (63.66) | 0 (0) |
| Not determined | 67 (24.01) | 7 (15.91) | 74 (22.91) | 185 (23.51) | 164 (100) |
| **Vaccination status^a^** |  |  |  |  |  |
| Unvaccinated | 241 (86.38) | 41 (93.18) | 282 (87.31) | 186 (23.63) | 21 (12.8) |
| Partial | 7 (2.51) | 0 (0) | 7 (2.17) | 35 (4.45) | 8 (4.88) |
| Full | 31 (11.11) | 3 (6.82) | 34 (10.53) | 332 (42.19) | 75 (45.73) |
| Booster | 0 (0) | 0 (0) | 0 (0) | 233 (29.61) | 60 (36.59) |
| **Underlying disease** |  |  |  |  |  |
| None | 266 (95.34) | 39 (88.64) | 305 (94.43) | - | - |
| Febrile seizures | 6 (2.15) | 2 (4.55) | 8 (2.48) | - | - |
| Solid Tumor | 3 (1.08) | 0 (0) | 3 (0.93) | - | - |
| Congenital heart diseases | 2 (0.72) | 0 (0) | 2 (0.62) |  |  |
| Leukemia | 0 (0) | 1 (2.27) | 1 (0.31) | - |  |
| Rett syndrome | 0 (0) | 1 (2.27) | 1 (0.31) |  | - |
| Crohn’s disease | 1 (0.36) | 0 (0) | 1 (0.31) |  |  |
| Allergic rhinitis | 1 (0.36) | 1 (2.27) | 2 (0.62) | - | - |
| **Clinical severity** |  |  |  |  |  |
| **Symptomatic** | **270 (96.77)** | **44 (100)** | **314(97.21)** | **675 (85.77)** | **-** |
| Mild | 270 (96.77) | 0 (0) | 270 (83.59) | - | - |
| Moderate | 0 (0) | 43 (97.73) | 43 (13.31) | - | - |
| Critical | 0 (0) | 1 (2.27) | 1 (0.31) | - | - |
| **Asymptomatic** | **9 (3.23)** | **0 (0)** | **9 (2.79)** | **112 (14.23)** | **-** |
| **Symptoms** |  |  |  |  |  |
| Fever | 263 (94.27) | 43 (97.73) | 306 (94.74) | - | - |
| Fever spike (℃) | 39.3±0.7 | 39.4±1.1 | 39.3±0.8 | - | - |
| Fever duration (days) | 2 (1-3) | 3 (1-3) | 2 (1-3) | - | - |
| Cough | 107 (38.35) | 25 (56.82) | 132 (40.87) | - | - |
| Nausea/vomiting/  diarrhea | 42 (15.05) | 11 (25) | 53 (16.41) | - | - |
| Stuffy nose | 27 (9.68) | 5 (11.36) | 32 (9.91) | - | - |
| Sore throat | 15 (5.38) | 4 (9.09) | 19 (5.88) | - | - |
| Loss of taste/smelling | 3 (1.08) | 1 (2.27) | 4 (1.24) | - | - |

^a^Partial vaccination was defined as an individual receiving only one-dose inactivated vaccine. Full vaccination was defined as an individual receiving two doses of inactivated SARS-CoV-2 vaccines for at least 2 weeks. Booster vaccination was defined as a fully vaccinated individual receiving an additional dose of inactivated vaccine for at least 14 days.

**Supplementary Table 3.** Estimates of the incubation period based on the analysis of 52 cases from 28 households.

| **Distribution** | **Parameters**  **[mean (SD)]** | **Mean**  **(days)** | **Quantiles**  **(0.025-0.975, days)** | **AIC** |
| --- | --- | --- | --- | --- |
| Gamma | Shape = 3.93 (1.20);  Rate =0.84 (0.29) | 4.67 | 1.25-10.3 | 84.53 |
| Weibull | Shape = 2.36 (0.43);  Scale = 5.19 (0.48) | 4.60 | 1.10-9.03 | 84.14 |
| Lognormal | Meanlog = 1.42 (0.11);  Sdlog = 0.57 (0.10) | 4.87 | 1.36-12.62 | 85.62 |

**Supplementary Table 4.** Estimates of the serial interval based on the analysis of 234 transmission pairs.

| **Distribution** | **Parameters^a^**  **[mean (SD)]** | **Mean**  **(days)** | **Quantiles**  **(0.025-0.975, days)** | **AIC** |
| --- | --- | --- | --- | --- |
| Gamma | Shape = 17.31 (0.17);  Rate =1.19 (0.01) | 4.10 | -1.95-11.74 | 107863.7 |
| Weibull | Shape = 4.49 (0.02);  Scale = 15.75 (0.03) | 3.87 | -3.56-10.56 | 106455.0 |
| Lognormal | Meanlog = 2.65 (0.002);  Sdlog = 0.30 (0.001) | 4.34 | -2.63-15.06 | 116566.8 |

^a^ We fitted the distribution with a shift parameter equals to 10.5 days allowing negative serial intervals

**Supplementary Table 5.** Estimates of the infectiousness profile based on the analysis of 234 transmission pairs.

| **Distribution** | **Parameters^a^**  **[mean (SD)]** | **% of presymptomatic transmission** | **AIC** |
| --- | --- | --- | --- |
| Gamma | Shape = 25.72 (0.25); Rate =1.35 (0.01) | 57.2% | 110909.0 |
| Weibull | Shape = 5.20 (0.02); Scale = 20.50 (0.03) | 53.8% | 112063.4 |
| Lognormal | Meanlog = 2.93 (0.001); Sdlog = 0.21 (0.001) | 57.9% | 113611.3 |

^a^ We fitted the distribution with a shift parameter equals to 19.5 days allowing presymptomatic transmission.

**
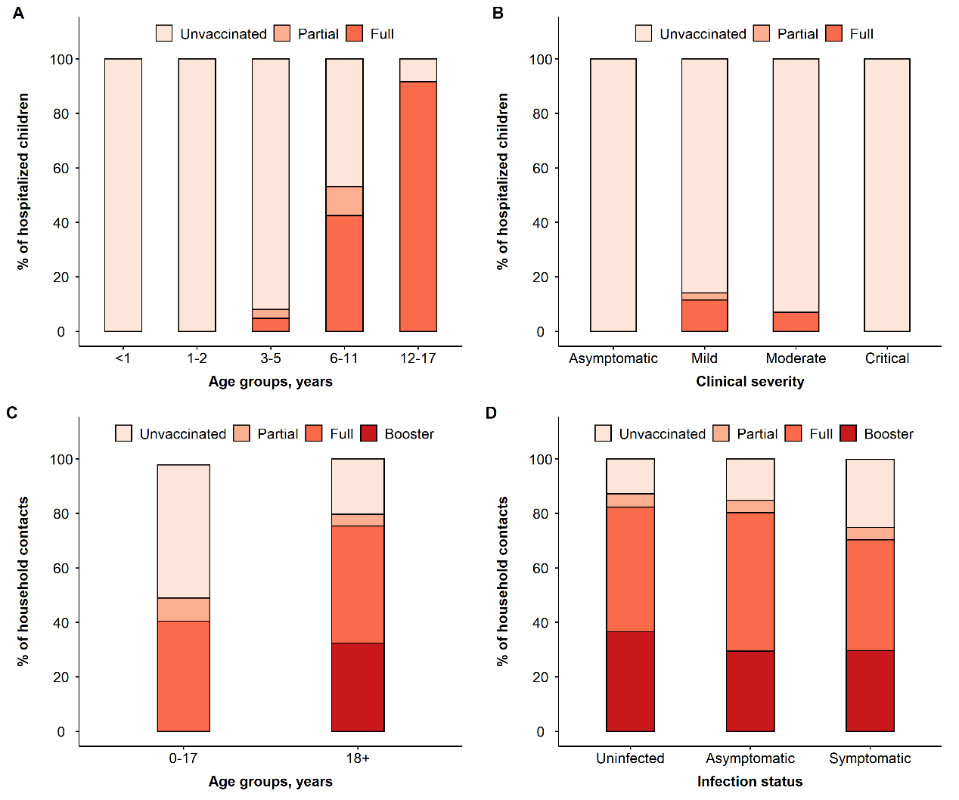
**

**Supplementary Fig. 1. Vaccination status of the 323 hospitalized pediatric cases and their 951 household members. (A).** Vaccination status of the 323 hospitalized pediatric cases by age. **(B).** Vaccination status of the 323 hospitalized pediatric cases by clinical severity. **(C).** Vaccination status of the 951 household members by age. **(D).** Vaccination status of the 951 household members by infection status.

**
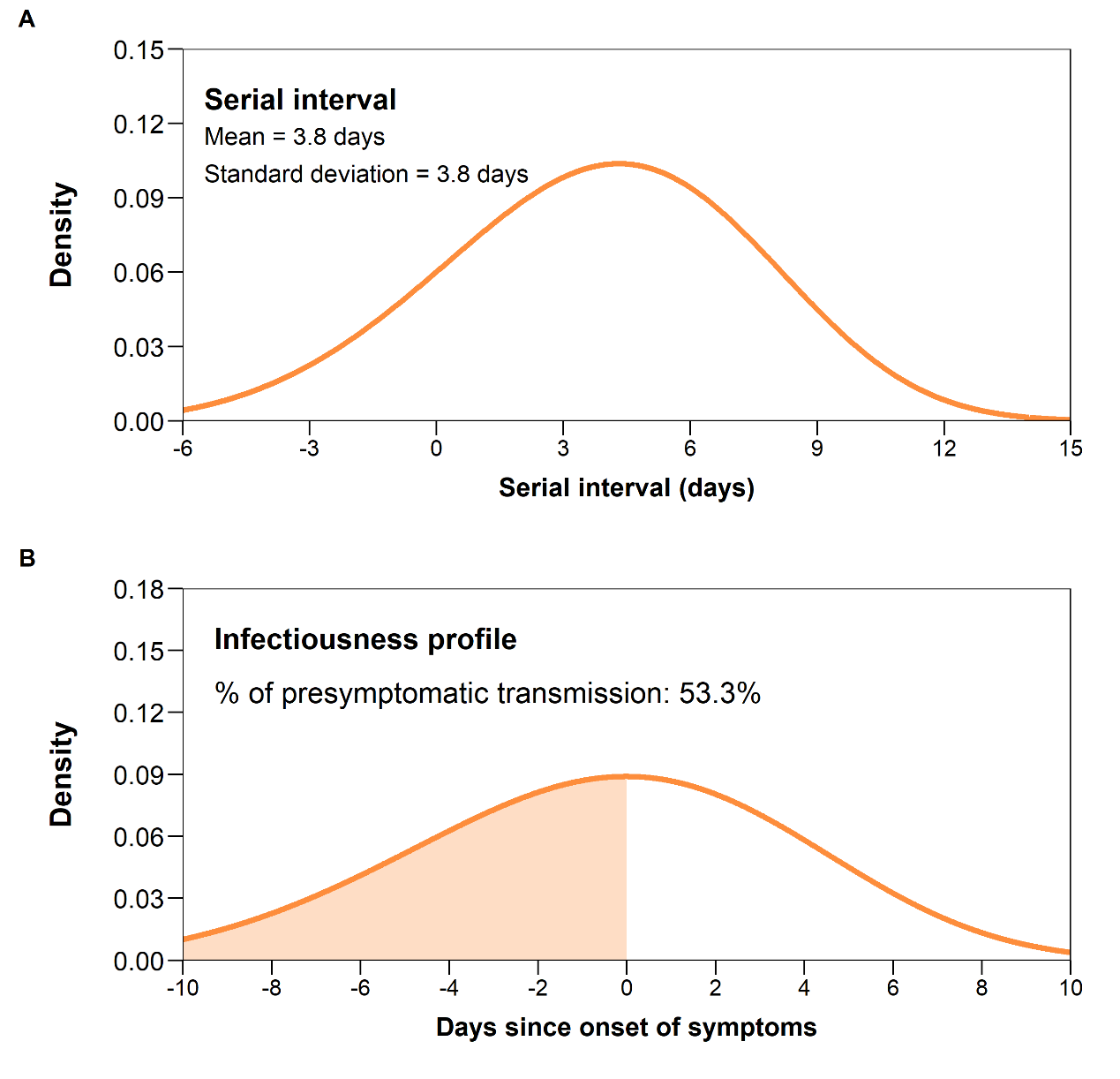
**

**Supplementary Fig. 2. Best fitted distributions of the serial interval and infectiousness profile since onset of symptoms** **(sensitivity analysis),** for the situation that all secondary cases of a household are from the same primary case, not accounting for the uncertainty caused by co-primary cases. **(A)** Estimated distribution of the serial interval based on the analysis of 159 transmission pairs. **(B)** Estimated infectiousness profile since onset of symptoms based on the analysis of 159 transmission pairs.


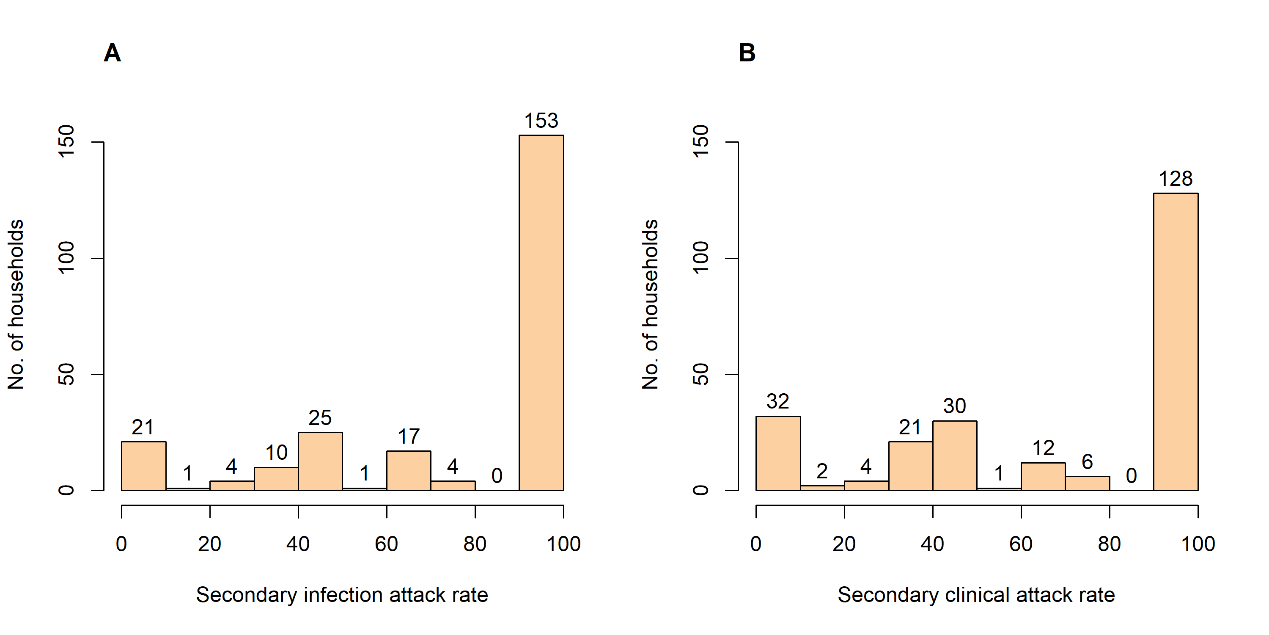


**Supplementary Fig. 3. The heterogeneity in terms of (A) secondary infection attack rates and (B) secondary clinical attack rates across households.**
